# Characteristics of ventilator-associated pneumonia due to hypervirulent *Klebsiella pneumoniae* genotype in genetic background for the elderly in two tertiary hospitals in China

**DOI:** 10.1186/s13756-018-0371-8

**Published:** 2018-08-03

**Authors:** Chao Liu, Jun Guo

**Affiliations:** 1 Department of Respiratory Medicine, Peking Union Medical College, Chinese Academy of Medical Sciences, China-Japan Friendship Hospital, Beijing, China; 2 Department of Respiratory Medicine, School of Clinical Medicine, Beijing Tsinghua Changgung Hospital, Tsinghua University, Beijing, China; 3 Department of Geriatric Respiratory Medicine, Chinese PLA General Hospital, Beijing, China

**Keywords:** *Klebsiella pneumoniae*, Hypervirulent, Aerobactin, Risk factor, ESBL-hvKp, Ventilator-associated pneumonia

## Abstract

**Background:**

Aerobactin is a critical factor for the hypervirulent *Klebsiella pneumoniae* (hvKp), but data for the aerobactin-positive genotype of hvKp in elderly persons with ventilator-associated pneumonia (VAP) is limited. The purpose of this study is to understand the risk factors and characteristics of the hvKp genotype for elderly patients with VAP.

**Methods:**

A retrospective study of 73 elderly patients with *Kp* was conducted from November 2008 to December 2017 in two tertiary hospitals. The clinical and microbiological data, including inflammatory reaction, nutritional status, antimicrobial susceptibility testing, string test, extended-spectrum-β-lactamase (ESBL) production, virulence-associated gene (capsular serotype-specific gene and rmpA/A2,magA,aerobaction) and multilocus sequence typing, of the hvKp group defined as aerobactin positive were compared with those of classic *Kp* strains.

**Results:**

Of 73 *Kp* isolates, 46.6% were hvKp. ST23 is highly prevalent in two hospitals but is not highly associated with hvKp in different hospitals. Additionally, ST23, ST37 and ST2906 are more likely to induce lethal VAP. Most hvKp strains are sensitive to common antibiotics, but the number of multidrug-resistant (MDR) hvKp is increasing. Importantly, 38.2% of hvKp isolates produced ESBLs. Hypermucoviscosity and virulence-associated genes (K1,magA and rmpA/A2) were highly clustered in the hvKp group (*P* < 0.001). Cancer (*P* = 0.004), digestive disease (*P* = 0.038) and surgery (*P* = 0.023) within 1 month are strongly associated with the VAP-hvKp group. The incidence of septic shock (*P* = 0.016) and Sequential Organ Failure Assessment (SOFA) scores (*P* < 0.001) are significantly higher in the hvKp group. Multivariate analysis indicated that cancer (odds ratio [OR] = 5.365) is an independent risk factor for VAP-hvKp infection.

**Conclusions:**

The morbidity for elderly patients with VAP due to hvKp is high. MDR-HvKp is emerging, which is a great challenge for clinical practice.

## Background

Ventilator-associated pneumonia (VAP) is the most frequent life-threatening nosocomial infection in critically ill patients [1, 2]. *Klebsiella pneumoniae* (Kp) is one of the most common gram-negative bacteria causing hospital infections, especially various notoriously fatal infections. Kp includes two distinct groups: hypervirulent (hvKp) and classical (cKp). One of the characteristics of cKp is acquiring an antibiotic-resistant gene. The other type is hvKp, which is traditionally defined as hypermucoviscosity by string test, inducing aggressive invasive community infection, bloodstream infection, and pyogenic liver abscesses (PLA) for immunocompetent ambulatory younger adults with no underlying diseases [3–6]. However, the definition of hvKp by string test is controversial [7, 8]. Previous studies showed that certain hypermucoviscous *K. pneumonia* (hmvKp) strains are not closely related to high virulence in in vitro and in vivo models [7, 8]. Thus, to differentiate hvKp from cKp by the hypermucoviscosity phenotype alone may be inappropriate [9, 10]. Recently, aerobactin, a dominant component of the siderophore system, has been established as a critical virulence factor for the hvKp genotype [3, 10, 11]. A previous multi-centre study in China targeting middle-aged patients, illustrated the clinical and molecular characteristics of hvKp (defined as aerobactin positive) infection [10]. However, the data for the hospital infection in the elderly with hvKp VAP, who commonly suffer from various underlying disease and nutritional complications, is rare.

Although most previous studies demonstrated that hvKp is sensitive to most antibiotics, which is prominently different from cKp strains, the multi-drug resistance (MDR) and extended-spectrum-β-lactamase (ESBL)-producing hvKp, especially those resistant to colistin and carbapenems, are emerging in China [12–14]. However, there are not enough referable data for the elderly with VAP due to antimicrobial-resistant hvKp.

To date, no data about the clinical and microbiological characteristics of VAP caused by the hvKp genotype in the elderly have been demonstrated. The aim of this study was to further investigate the clinical characteristics of elderly patients with hvKp infection and microbiological features and the epidemiology of multi-centre hvKp. Therefore, we conducted a retrospective study in two tertiary hospitals focusing on the genotype of hvKp (defined as aerobactin positive) in China.

## Methods

### Patients

A retrospective study was conducted on 73 *Kp* culture-positive patients diagnosed from November 2008 to December 2017 at two tertiary hospitals: Beijing Tsinghua Changgung Hospital and Chinese PLA General Hospital. These two hospitals are located in Beijing, and the capacity of the hospitals is more than 800 beds. Patients currently receiving mechanical ventilation were included in this study. The definition of VAP is according to the 2016 ATS guideline [15]. Standard bronchoalveolar lavage and aspiration were achieved. The clinical characteristics, including underlying disease, infection type, nutritional status, mortality in 30 days, and sequential organ failure assessment (SOFA) were collected. The primary outcome of this study was to investigate the 30-day mortality of hvKp infection in the elderly with VAP compared with that of the cKp group. Additionally, the white blood cell count (WBC) and neutrophil percentage (NEU%) were applied as primary inflammatory factors. Total protein (TP) and albumin (ALB) were used to evaluate the nutrition status. The main inclusion criteria were 1) the definition of the elderly as 65 years old or older (≥65 year) and 2) at least one *K. pneumoniae*-positive culture. The exclusion criteria was 1) insufficient clinical data or bacterial strain sample storage and 2) cases of co-infection.

### Clinical *Kp* strains

All isolates were stored at − 80 °C and identified by the API 20 NE system and the VITEK II system. Further species identification was confirmed by 16S rRNA gene sequencing. HvKp is defined as aerobactin positive. The hypermucoviscous phenotype was detected by the string test as described previously [16].

### Antimicrobial susceptibility testing and phenotypic detection of ESBLs

Antimicrobial susceptibility testing was performed, and the results were interpreted according to 2017 Clinical and Laboratory Standards Institute (CLSI) guidelines. The antibiotics include amikacin, gentamicin, tobramycin, Sulbactam/sulbactam, aztreonam, cefazolin, cefepime, ceftriaxone, ceftazidime, ciprofloxacin, levofloxacin, piperacillin/tazobactam, and trimethoprim/sulfamethoxazole. ESBL was detected by an agar dilution test using ceftazidime and cefotaxime combined with clavulanate according to the CLSI guideline [10]. The definition of an MDR strain is resistance to three or more different antimicrobial categories, as described previously [17]. The carbapenem-resistant (CR) phenotype is defined as resistant to both imipenem and meropenem.

### Detection of virulence-associated gene

Genomic DNA of all *Kp* isolates was extracted. *RmpA, rmpA2, magA*, *aerobactin* and the capsular serotype-specific (*cps*) genes (*K1, K2, K5, K20, K54,* and *K57*) were detected by polymerase chain reaction (PCR) as described previously [10, 18–20]. The primers are listed in Table 1.

**Table 1:** Primers

Name	sequence
*rmpA*	
Forward	5-ACTGGGCTACCTCTGCTTCA-3
Reverse	5-CTTGCATGAGCCATCTTTCA-3
*rmpA2*	
Forward	5-CTTTATGTGCAATAAG-GATGTT-3
Reverse	5-CCTCCTGGAGAGTAAGCATT-3
*magA*	
Forward	5-GGTGCTCTTTACATCATTGC-3
Reverse	5-GCAATGGCCATTTGCGTTAG-3
*aerobactin*	
Forward	5-GCATAGGCGGATACGAACAT-3
Reverse	5-CACAGGGCAATTGCTTACCT-3
*K1*	
Forward	5-GTAGGTATTGCAAGCCATGC-3
Reverse	5-GCCCAGGTTAATGAATCCGT-3
*K2*	
Forward	5-GGAGCCATTTGAATTCGGTG-3
Reverse	5-TCCCTAGCACTGGCTTAAGT-3
*K5*	
Forward	5-GCCACCTCTAAGCATATAGC-3
Reverse	5-CGCACCAGTAATTCCAACAG-3
*K20*	
Forward	5-CCGATTCGGTCAACTAGCTT-3
Reverse	5-GCACCTCTATGAACTTTCAG-3
*K54*	
Forward	5-CATTAGCTCAGTGGTTGGCT-3
Reverse	5-GCTTGACAAACACCATAGCAG-3
*K57*	
Forward	5-CGACAAATCTCTCCTGACGA-3
Reverse	5-CGCGACAAACATAACACTCG-3
*rpoB*	
Forward	5-GGCGAAATGGCWGAGAACCA-3
Reverse	5-GAGTCTTCGAAGTTGTAACC-3
*gapA*	
Forward	5-TGAAATATGACTCCACTCACGG-3
Reverse	5-CTTCAGAAGCGGCTTTGATGGCTT-3
*mdh*	
Forward	5-TGAAATATGACTCCACTCACGG-3
Reverse	5-CTTCAGAAGCGGCTTTGATGGCTT-3
*pgi*	
Forward	5-GAGAAAAACCTGCCTGTACTGCTGGC-3
Reverse	5-CGCGCCACGCTTTATAGCGGTTAAT-3
*phoE*	
Forward	5-ACCTACCGCAACACCGACTTCTTCGG-3
Reverse	5-TGATCAGAACTGGTAGGTGAT-3
*infB*	
Forward	5-CTCGCTGCTGGACTATATTCG-3
Reverse	5-CGCTTTCAGCTCAAGAACTTC-3
*tonB*	
Forward	5-CTTTATACCTCGGTACATCAGGTT-3
Reverse	5-ATTCGCCGGCTGRGCRGAGAG-3

### Multilocus sequence typing (MLST) for Kp

Seven housekeeping genes (*gapA, mdh, phoE, tonB, infB, pgi and rpoB*) were detected by PCR according to the MLST website (*http://bigsdb.pasteur.fr/klebsiella/ klebsiella.html*) (Table 1). Allelic profiling and sequence types (STs) were also performed using the above website. Moreover, to further distinguish the relationship among different STs, phylogenetic analysis of the seven spliced housekeeping genes for these isolates that contributed to mortality was performed by the neighbour-joining method (MEGA 6.0). The common STs, including ST1, ST11, ST37, ST258 and ST412, were used as a reference.

### Statistical analysis

SPSS software (version 20.0) was employed for data analysis. Measurement data was assessed as the means ± standard deviation (SD). The count data were analysed as percentages. Continuous variables were analysed by Student’s *t*-tests and the Wilcoxon rank-sum tests. Categorical variables were analysed by the χ^2^ or Fisher’s exact tests. Univariate logistic regression analysis was performed for the risk factors. A multivariable logistic regression analysis was conducted for independent risk factors (the variables with *P*<0.05 were included). All tests were 2-tailed. A *p*-value<0.05 was considered significant.

## Results

### Clinical characteristics

Seventy-three *Kp* culture-positive patients were diagnosed at the two hospitals from November 2008 to December 2017 (Table 2). Thirty-four (46.6%) strains were hvKp, and 37 (50.7%) were hmvKp. Most of the patients (67, 91.8%) presented with sepsis, and 24 patients (32.9%) were diagnosed with septic shock. Sixty-six (90.4%) were males, and the mean age of patients in this study was 84.96 ± 8.33 years. Patients with cancer (38.2% versus 7.7%; *P* = 0.004), surgery history within 1 month (23.5% versus 5.1%; *P* = 0.023) and digestive diseases (29.4% versus 10.3%; *P* = 0.046) were more likely to be infected with hvKp. Although host responsibility (WBC and NEU%) and nutritional status (TP and ALB) were not significantly different at the primary endpoint (30-day mortality), the SOFA scores of the patients with hvKp were notably higher at 30-day mortality (8.94 ± 3.03 vs 6.62 ± 2.09, *P* = 0.000) (Table 2).

**Table 2:** Clinical features of patients with VAP due to hvKp

Characteristic	HvKp(34)	cKp(39)	*P* value
Basic demographics			
Age	83.06 ± 8.55	86.62 ± .87	0.068
Male	31 (91.2%)	35 (89.7%)	1.000
Underlying diseases			
Pulmonary disease	31 (91.2%)	32 (82.1%)	0.321
Diabetes	19 (55.9%)	18 (46.2%)	0.407
Cardiovascular disease	12 (35.3%)	17 (43.6%)	0.470
Cerebrovascular disease	11 (32.4%)	15 (38.5%)	0.587
Cancer	**13 (38.2%)**	**3 (7.7%)**	**0.004**
Surgery within 1 mo	**8 (23.5%)**	**2 (5.1%)**	**0.023**
Digestive disease	**10 (29.4%)**	**4 (10.3%)**	**0.038**
Infection type			
Sepsis	30 (88.2%)	37 (94.9%)	0.408
Septic shock	**16 (47.1%)**	**8 (20.5%)**	**0.016**
Host responsibility			
WBC	12.57 ± 4.47	11.68 ± 4.36	0.397
NEU%	78.99 ± 9.54	77.13 ± 7.14	0.344
Nutrition status			
TP	63.38 ± 6.34	61.88 ± 6.02	0.304
ALB	33.33 ± 3.75	32.51 ± 3.39	0.326
SOFA score	**8.94 ± 3.03**	**6.62 ± 2.09**	**0.000**
Infection occurred in ICU	11 (32.4%)	10 (25.6%)	0.527
Mortality in 30 days	14 (41.2%)	14 (35.9%)	0.644

*TP* total protein, *ALB* albumin, *WBC* white blood cell count, *NEU%* neutrophils percentage

### Genetic and phenotype characteristics: hvKp vs cKp

It is noted that hypermucoviscosity was strongly clustered in the hvKp group (*P* < 0.001). Our results showed that *K1, rmpA, rmpA2* and *magA* were highly clustered in hvKp (*P* < 0.001), but *K2*, *K5, K20, K54,* and *K57* were not associated with hvKp (*P* = 0.073, 0.213, 1.000, 0.096 and 0.849, respectively). Additionally, there is no isolate in the cKp group with K5 or K54 (Table 3).

**Table 3:** Microbiological features of patients with VAP due to hvKp

Characteristic	HvKp(34)	cKp(39)	*P* value
K serotype			
K1	**17 (50.0%)**	**1 (2.6%)**	**0.000**
*K2*	7 (2.9%)	2 (5.1%)	0.073
*K5*	2 (5.9%)	0 (0%)	0.213
*K20*	2 (5.9%)	2 (5.1%)	1.000
*K54*	3 (8.8%)	0 (0%)	0.096
*K57*	4 (11.8%)	3 (7.7%)	0.849
rmpA	**27 (79.4%)**	**4 (10.3%)**	**0.000**
rmpA2	**28 (82.4%)**	**5 (12.8%)**	**0.000**
magA	**30 (88.2%)**	**17 (43.6%)**	**0.000**
Hypermucoviscosity	**29 (85.3%)**	**8 (20.5%)**	**0.000**

### Antimicrobial resistance analysis and detection rate of ESBL-producing *Kp* isolates

All Kp strains were resistant to Sulbactam. Most of the hvKp isolates represented a higher antimicrobial sensitivity rate to most of the antibiotics than cKp, except cefepime, ceftriaxone, ciprofloxacin, levofloxacin, imipenem, meropenem, and amikacin (Table 4). Although the detection rate of MDR (41.2% vs 74.4%, *P* = 0.001) and ESBLs (38.2% vs 69.2%, *P* = 0.001) was significantly lower than cKp, it is noted that 41.2% (14/34) of hvKp isolates were MDR and 38.2% (13/34) of hvKp isolates were ESBL-producing. Moreover, 8 isolates were identified as CR-hvKp.

**Table 4:** Antibiotics resistance: hvKp vs cKp

Antibiotic agent	HvKp(34)	cKp(39)	*P* value
MDR	**14 (41.2%)**	**29 (74.4%)**	**0.004**
ESBLs	**13 (38.2%)**	**27 (69.2%)**	**0.008**
Ampicillin	34 (100%)	39 (100%)	NA
Amikacin	6 (17.6%)	12 (30.8%)	0.194
Gentamicin	**8 (23.5%)**	**21 (53.8%)**	**0.008**
Ampicillin/Sulbactam	**13 (38.2%)**	**27 (69.2%)**	**0.008**
Aztreonam	**10 (29.4%)**	**21 (53.8%)**	**0.035**
Cefazolin	**14 (41.2%)**	**28 (71.8%)**	**0.008**
Cefotetan	**8 (23.5%)**	**16 (41.0%)**	**0.112**
Cefepime	9 (26.5%)	18 (46.2%)	0.082
Ceftriaxone	13 (38.2%)	23 (59.0%)	0.077
Ceftazidime	**10 (29.4%)**	**23 (59.0%)**	**0.011**
Ciprofloxacin	10 (29.4%)	20 (51.3%)	0.058
Levofloxacin	9 (26.5%)	19 (48.7%)	0.051
Trimethoprim/Sulfamethoxazole	**7 (20.6%)**	**22 (56.4%)**	**0.002**
Piperacillin/Tazobactam	**8 (23.5%)**	**18(46.2%)**	**0.044**
Imipenem	8 (23.5%)	10(25.6%)	0.835
Meropenem	9 (26.5%)	10(25.6%)	0.936
Tobramycin	**10 (29.4%)**	**21(53.8%)**	**0.027**

### Risk factors for VAP-hvKp

Univariate regression analysis showed that cancer (odds ratio [OR] = 7.429), digestive diseases (OR = 3.646) and surgery history within 1 month (OR = 5.692) were risk factors for hvKp infection. Multivariate analysis revealed that cancer history (OR = 5.365) was an independent risk factor for hvKp infection (Table 5).

**Table 5:** Risk factor for hvKp vs cKp

Variable	Univariate OR (95% CI)	*P* value	Multivariate OR (95% CI)	*P* value
Infection occurred in ICU	1.387 (0.502–3.832)	0.528		
Male	1.181 (0.245–5.694)	0.836		
Pulmonary diseases	2.260 (0.536–9.539)	0.267		
Diabetes	1.478 (0.586–3.725)	0.408		
Cardiovascular disease	0.706 (0.274–1.818)	0.471		
Cerebrovascular disease	0.765 (0.291–2.010)	0.587		
Cancer	**7.429 (1.895–29.114)**	**0.004**	**5.365 (1.199–24.007)**	**0.028**
Surgery within 1 mo	**5.692 (1.117–29.013)**	**0.036**		
Digestive diseases	**3.646 (1.023–12.990)**	**0.046**	3.713 (0.970–14.212)	0.055

### MLST genotypic analysis

**Fig. 1:**
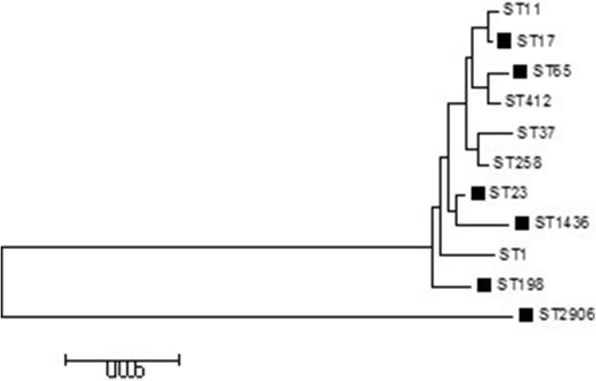
Neighbour-joining dendrogram of concatenated sequences of seven housekeeping genes from the MLST database for prevalent STs in our study and the common STs in the world. (Black solid box represents death in our study)

New STs were not detected among the 73 Kp isolates. The most prevalent ST in this study was ST23 (*n* = 15; 20.5%), followed by ST412 (*n* = 5; 6.8%), ST17 (*n* = 4; 5.5%), ST37 (*n* = 3; 4.1%), and ST2906 (*n* = 3; 4.1%). The above STs accounted for 41.1% (30/73) of the total strains. Among the prevalent STs, 73.3% ST23 (11/15), 60.0% ST412 (3/5), and 100% ST17 (4/4) were hvKp. The detailed features of patients with prevalent hvKp are shown in Table 6. The CR-hvKp distributed in ST17 (*n* = 2), ST23 (*n* = 1), ST347 (*n* = 1), ST412 (*n* = 1), ST2874 (*n* = 1), and ST2905 (*n* = 1). The most common clone complex (CC) of the CR-hvKp was CC17 (*N* = 2). The phylogenetic tree demonstrated the relationship between the prevalent STs in our study and the common STs in the world (Fig. 1).

**Table 6:** Features of patients with prevalent hvKp(n ≥ 3)

	ST2906(3)		ST37(3)		ST17(4)		ST412(5)		ST23(15)	
	*N*^a^ = 0	*N*^b^ = 3	*N*^a^ = 2	*N*^b^ = 1	*N*^a^ = 2	*N*^b^ = 2	*N*^a^ = 2	*N*^b^ = 3	*N*^a^ = 10	*N*^b^ = 5
K serotype										
*K1*	0	0	0	0	0	2	0	1	9	0
*K2*	0	1	1	1	0	2	0	1	0	0
*K5*	0	1	0	0	0	0	0	0	0	1
*K20*	0	1	0	0	0	0	0	0	0	1
*K54*	0	0	0	0	0	1	0	0	0	0
*K57*	0	0	1	0	2	1	1	0	0	0
*rmpA*	0	0	1	0	2	2	2	0	10	0
*rmpA2*	0	0	1	0	2	2	2	1	9	0
*magA*	0	0	2	0	2	2	2	1	9	1
Hmv	0	0	0	0	2	2	2	1	9	0
hvKp (aerobactin)	0	1	0	0	2	2	2	1	10	1
Sepsis Shock	0	3	0	1	2	1	0	1	8	1
Died in 30 Days	0	3	2	1	2	0	0	0	8	1

*Hmv* hypermucoviscosity, *a* and *b* different hospital

## Discussion

To our knowledge, this is the largest multi-centre study focusing on elderly patients with VAP due to hvKp in China. Nearly half (46.6%, 34/73) of the hvKp-induced VAP in the elderly patients occurred among mechanically ventilated patients. The hvKp group showed a higher severity of disease than did the cKp group. The primary virulence factors, such as K1, rmpA/A2, and magA, were highly clustered in the hvKp group. ST23 was the most prevalent ST in two hospitals. However, the ST23 isolated in different hospitals was not highly associated with the aerobactin genetic background or hypermucoviscous phenotype, and it was concluded that just relying on STs to identify hvKp may be not appropriate. In our study, 50.7% of *K. pneumonia* isolates were identified as hypermucoviscous by the string test experiment, which is higher than that in a previous retrospective study conducted in a single centre in China, with a prevalence of 33% [16]. Thus, plasmid type, biofilm production, serotypes and the ability to induce inflammatory factors may be needed to further define hvKp. The prevalence of hvKp may be incorrectly estimated because of the lack of definite and objective diagnostic methods.

Aerobactin was considered a potential virulence trait for the hvKp genotype in in vitro and in vivo models [3]. Importantly, previous studies showed that the hvKp genotype (aerobactin-positive) often induced more serious invasive infection [10, 11, 21]. Previous studies reported that the detection rate of hvKp ranged from 4.5 to 37.8% in various clinical specimens [7, 10, 16, 19, 22]. In this study, 34 isolates (46.6%) were defined as hvKp due to aerobactin-positivity, which is the highest incidence for hospital-acquired infection (HAI) caused by the hvKp genotype. It is noted that the detection of the hvKp genotype in this study was also higher than that in previous multicentre studies(37.8%) based on adults [10] and a single centre retrospective study (28.6%) focusing on VAP [23]. It is concluded that hvKp may emerge as a major pathotype for elderly with VAP in the two hospitals.

Additionally, capsular serotype-specific genes are essential factors for *K. pneumoniae*. To date, various types of K-antigen have been reported [20, 24, 25]. In Asia, K1 and K2 are the most important elements, inducing severe infections. However, they are not the unique trait for hvKp [26–29]. Previous studies demonstrated that K1 distribution in hvKp ranged from 23 to 98% in various specimens in various populations [29–33]. The detection rate of K2 in hvKp ranged from 10 to 46% [16, 31, 33]. Moreover, a previous single centre study focusing on VAP showed that *K1* and *K2* among hvKp accounted for 42.9 and 21.4%, respectively [23]. The above data implied that K1 might be highly associated with hvKp compared with K2. In our study, half of the hvKp (50%) isolates were associated with *K1*, and *K2* was detected in 7 hvKp isolates (2.9%), which was consistent with previous studies. In addition to *K1* and *K2*, there were no significant difference between hvKp and cKp in *K5, K20, K54,* and *K57* due to small specimens. Moreover, *rmpA/rmpA2* and *magA* genes responsible for the hypermucoviscous phenotype were proposed as virulence factors in addition to major cps *K1/K2* [5, 19, 30, 34]. In our study, 2 hvKp isolates inserted *rmpA* did not show hypermucoviscosity. It is implied that there might exist other regulatory mechanisms for hypermucoviscosity. Importantly, our results are consistent with previous study: most of the dominant virulence-associated factors (*K1*, *rmpA/A2* and *magA*) and hypermucoviscosity are highly clustered in the hvKp group [10].

Although there was no significant difference in sepsis incidence and mortality between the two VAP groups, the incidence of sepsis shock and SOFA score was higher than those in the cKp group. Thus, to further understand the risk factors for hvKp, timely and appropriately prevention is essential. Our results showed that elderly patients with cancer, digestive disease and surgery history within 1 month were more likely to be infected with hvKp. Moreover, cancer history was an independent risk factor for VAP-hvKp infection in the elderly, and cancer patients should be monitored more intensively to prevent infection due to immunocompromise, which may also be a critical factor for VAP-hvKp [10]. Previous multi-centre study of the hvKp genotype based on adults in China concluded that diabetes and cancer were independent risk factors for hvKp [10]. However, the incidence of diabetes (50.7%, 37/73) in our study is far more than previous multi-centre study focused on adults (17.0%, 39/230) [10]. This may be the characteristics of the elderly who often suffer from various underlying diseases. Additionally, a previous study concluded that major histocompatibility complex (MHC) variants, nutritional status, and the gut microbiota might be potential host factors for improving the understanding of the hypervirulence phenomenon [9]. Although the incidence of septic shock and SOFA score in our study were higher than those of the cKp group, there was no significant difference in the nutritional status (TP and ALB) and inflammatory reaction (WBC and NEU%), which was consistent with a previous study [23]. Thus, other inflammatory factors may be needed to screen as a marker for elderly patients with VAP-hvKp.

Previous studies revealed that most cKp and antimicrobial-resistant patterns were overlapping, which is not frequent in hvKp [10, 16]. In this study, most of the hvKp isolates were sensitive to most of the above antibiotics. However, in the hvKp group, the number of ESBL-hvKp (41.2%) and MDR-hvKp (38.2%) is significantly higher than that in previous studies [10, 23]. It is alarming that MDR-hvKp may prefer to induce VAP in the elderly. Moreover, eight CR-hvKp isolates were identified as another “superbug”. Taken together, these data revealed that MDR-hvKp is emerging among elderly patients with VAP, which needs to be confirmed by further investigations in larger populations. The CR-hvKp isolates were not detected in the nosocomial environment by routine nosocomial infection surveillance. Additionally, the composition of the gut microbiota was unclear because anal swabs were not applied for nosocomial infection surveillance. Previous study suggested that wards previously infected with CR-hvKp should be disinfected and left unoccupied for more than 2 weeks [12]. It may be a good choice to prevent fatal outbreaks of infection, especially in critically ill and immunocompromised elderly patients with mechanical ventilation.

The main limitation in our study is that it is a retrospective study from over 10 years. Although this study included two large tertiary hospitals, fewer patients with VAP-hvKp were included. However, this study is the largest cohort to investigate the clinical and microbiological characteristics of elderly patients with VAP-hvKp to now. Additionally, most of the key inflammatory factors, nutrition statue markers and environmental samples were not achieved. Moreover, the expression of aerobactin is unclear. Whole genome sequencing, transcriptomics and proteomics may be needed for further study to identify genetic expression.

## Conclusions

HvKp is emerging as a common pathogen of VAP in the elderly in China. There is various epidemiology of VAP-hvKp in different hospitals. Although STs may not be associated with hvKp, ST23, ST37 and ST2906 are more likely to induce poor prognosis. Although the definition of hvKp is still controversial, the hvKp genotype is more likely to cause septic shock and a higher SOFA score. The emerging MDR-hvKp, especially CR-hvKp, will be a “superbug”, which is a great challenge for clinicians. It is essential to enhance clinical awareness and infection surveillance for various hvKp infections, especially in immunocompromised elderly patients.
